# Measuring socioeconomic status in multicountry studies: results from the eight-country MAL-ED study

**DOI:** 10.1186/1478-7954-12-8

**Published:** 2014-03-21

**Authors:** Stephanie R Psaki, Jessica C Seidman, Mark Miller, Michael Gottlieb, Zulfiqar A Bhutta, Tahmeed Ahmed, AM Shamsir Ahmed, Pascal Bessong, Sushil M John, Gagandeep Kang, Margaret Kosek, Aldo Lima, Prakash Shrestha, Erling Svensen, William Checkley

**Affiliations:** 1 Fogarty International Center, National Institutes of Health, Bethesda, USA; 2 Program in Global Disease Epidemiology and Control, Department of International Health, Bloomberg School of Public Health, Johns Hopkins University, Baltimore, USA; 3 Science Division, Foundation for the National Institutes of Health, Bethesda, USA; 4 Division of Women and Child Health, Aga Khan University, Karachi, Pakistan; 5 Division of Nutrition and Food Security, International Centers for Diarrheal Disease Research, Matlab, Bangladesh; 6 HIV/AIDS and Global Health Research Programme, University of Venda, Thohoyandou, South Africa; 7 Christian Medical College, Vellore, India; 8 Clinical Research Unit and Institute of Biomedicine, Federal University of Ceara, Fortaleza, Brazil; 9 Institute of Medicine, Tribhuvan University, Kathmandu, Nepal; 10 Centre for International Health, University of Bergen, Bergen, Norway; 11 Haydom Lutheran Hospital, Haydom, Tanzania

**Keywords:** Socioeconomic status, Child growth, Classification, Measurement

## Abstract

**Background:**

There is no standardized approach to comparing socioeconomic status (SES) across multiple sites in epidemiological studies. This is particularly problematic when cross-country comparisons are of interest. We sought to develop a simple measure of SES that would perform well across diverse, resource-limited settings.

**Methods:**

A cross-sectional study was conducted with 800 children aged 24 to 60 months across eight resource-limited settings. Parents were asked to respond to a household SES questionnaire, and the height of each child was measured. A statistical analysis was done in two phases. First, the best approach for selecting and weighting household assets as a proxy for wealth was identified. We compared four approaches to measuring wealth: maternal education, principal components analysis, Multidimensional Poverty Index, and a novel variable selection approach based on the use of random forests. Second, the selected wealth measure was combined with other relevant variables to form a more complete measure of household SES. We used child height-for-age Z-score (HAZ) as the outcome of interest.

**Results:**

Mean age of study children was 41 months, 52% were boys, and 42% were stunted. Using cross-validation, we found that random forests yielded the lowest prediction error when selecting assets as a measure of household wealth. The final SES index included access to improved water and sanitation, eight selected assets, maternal education, and household income (the WAMI index). A 25% difference in the WAMI index was positively associated with a difference of 0.38 standard deviations in HAZ (95% CI 0.22 to 0.55).

**Conclusions:**

Statistical learning methods such as random forests provide an alternative to principal components analysis in the development of SES scores. Results from this multicountry study demonstrate the validity of a simplified SES index. With further validation, this simplified index may provide a standard approach for SES adjustment across resource-limited settings.

## Introduction

Socioeconomic status (SES) is a theoretical construct encompassing individual, household, and/or community access to resources. It is commonly conceptualized as a combination of economic, social, and work status, measured by income or wealth, education, and occupation, respectively [1,2]. SES has been linked to a wide range of health-related exposures and outcomes, including child undernutrition, chronic disease, and infection [2,3]. In a review of risk factors for adverse outcomes in child cognitive development, Walker and colleagues [4] conceptualized poverty as underlying more proximal psychological and biological risk factors, including maternal depression and nutrient deficiencies. More recently, researchers have highlighted connections between childhood SES and lifetime health outcomes, such as heart disease and chronic obstructive pulmonary disease [5].

Literature on SES measurement distinguishes between wealth, or accumulated financial resources, and income, a measure of shorter-term access to capital [2]. Researchers have identified challenges in collecting income data, particularly in low-income settings, due to monthly fluctuations, informal work, and reporting biases [6]. Recent empirical work has drawn attention to the approach of supplementing or replacing information on income with direct measures of wealth, such as household assets [7]. Perhaps the most widespread approach to direct measurement of household wealth is that used by the Demographic and Health Surveys (DHS), implemented in more than 90 countries since 1984 [8]. Using nationally representative data from India, Filmer and Pritchett [7] created an index based on household ownership of assets and housing materials to serve as a proxy for wealth. The resulting index was internally valid and coherent, and robust to the choice of assets. Using additional data sets from Indonesia, Nepal, and Pakistan, they further argued that a composite asset index is as reliable as data on household consumption and is less subject to measurement error [7]. Their statistical approach, using principal components analysis (PCA), has since been adapted to create a household wealth index in each DHS dataset [8]. Concerns about this approach include its over-representation of urban settings, and its failure to distinguish between the poorest of the poor, particularly in rural areas [9]. Furthermore, this approach requires lengthy surveys of household assets. Several studies have found that rapid wealth appraisals requiring as few as four survey questions perform as well as the DHS wealth index in categorizing households [10] and predicting mortality [11].

Multicountry studies pose an added challenge to measuring SES. While approaches focused on asset ownership are often sufficient for homogenous populations, studies in more diverse populations must explore whether variables that measure SES, such as ownership of specific assets, have the same meaning across populations. The DHS wealth index is derived using country-specific data rather than globally pooled data. One result is that a household in the poorest wealth quintile in Egypt might be wealthier than a household in the richest wealth quintile in Ethiopia. Therefore, controlling for SES in pooled analyses using this approach, either by raw score or wealth quintile, is inappropriate. The United Nations Development Programme (UNDP) sought to overcome the challenge of comparing household SES across countries through the Multidimensional Poverty Index (MPI), introduced as an experimental measure in the 2010 Human Development Report. The MPI includes three equally weighted dimensions of household SES: education (years of schooling, school attendance), health (child mortality, nutrition), and standard of living (household attributes, asset ownership) [12].

The Malnutrition and Enteric Infections: Consequences for Child Health and Development (MAL-ED) study seeks to explore relationships between early exposures to malnutrition and enteric infections and their consequences for child growth and cognitive development across eight sites. Geographic, cultural, and socioeconomic differences between these sites present an added challenge to developing a measure of SES that is relevant in all sites. We sought to compare different approaches to measuring SES in resource-limited settings, and provide guidance for measuring SES accurately and simply in epidemiologic studies of diverse populations.

## Materials and methods

### Study setting

This study took place at the eight field sites of the MAL-ED study (see Table 1). Study sites are located in a mix of rural, urban, and peri-urban areas of: Dhaka, Bangladesh (BGD); Fortaleza, Brazil (BRF); Vellore, India (INV); Bhaktapur, Nepal (NEB); Naushahro Feroze, Pakistan (PKN); Loreto, Peru (PEL); Venda, South Africa (SAV); and Haydom, Tanzania (TZH). Sites used a standardized protocol for data collection.

**Table 1:** Description of MAL-ED study sites and mean WAMI scores

** Country**	**Urban/rural**	**Site description**	**Mean (SD) WAMI score**
Brazil	Urban	Parque Universitário is an urban community inhabited by poor and middle class families. The community has approximately 33,000 people with 12% less than 5 years old. Of 288 children ≤3 years old, 31% have < -1 and 9% < -2 HAZ.	0.80 (0.08)
Peru	Peri-urban	The site is peri-urban with an economic base in agriculture, extraction of forest products, and fishing.	0.71 (0.11)
South Africa	Rural	The Dzimauli site is rural and mountainous, characterized by agricultural livelihood, low socioeconomic status, and poor infrastructure with waterfalls and many rivers across the villages. It is situated 25 km from the central business district.	0.70 (0.16)
Nepal	Peri-urban	The study site Bhaktapur Municipality and adjoining villages are peri-urban areas, with safe drinking water and toilet facilities. The main economic base is agriculture.	0.69 (0.12)
Bangladesh	Urban	Mirpur, an underprivileged community in Dhaka, is inhabited by poor and middle-class families. Residential and sanitary conditions are typical of any congested urban settlement. The investigators have ongoing research activities in the area.	0.55 (0.12)
Pakistan	Rural	The Molhan study site in district Naushahro Feroze is in the southern Sindh province. The site is surrounded by fertile plains near the Indus river, predominantly rural communities, agricultural occupations, low socioeconomic class, and poor infrastructure, including mud houses.	0.52 (0.17)
India	Urban	The study site is situated in a slum area in Vellore, which is a small city in Tamil Nadu in southern India. It is predominantly inhabited by poor families. The major occupation is manual labor in the market or construction work.	0.43 (0.10)
Tanzania	Rural	The Haydom area is ethnically and geographically diverse, situated at approximately 1700 meters above sea level and 300 kilometers from the nearest urban center. The study population is mainly agro-pastoralists.	0.22 (0.11)
		OVERALL	0.58 (0.22)

The WAMI score (range 0 to 1) measures household socioeconomic status, including access to improved Water/sanitation, Assets, Maternal education, and Income.

### Study design

Prior to beginning the ongoing cohort study, we conducted a cross-sectional feasibility study to identify the optimal approach to measuring household SES. We administered a standardized survey including demographic, socioeconomic status, and food insecurity questions to 100 households in each of the eight field sites between September 2009 and August 2010. Households were randomly selected from census results collected within the previous year at each site. Households were eligible to participate if they were located within the MAL-ED catchment area and if a child aged 24 to 60 months lived in the household. In households with multiple children in this age range, we randomly selected only one eligible child. Data collection lasted two to four weeks in each site. We obtained ethical approval from the Institutional Review Boards at each of the participating research sites, the Johns Hopkins Bloomberg School of Public Health (Baltimore, USA) and the University of Virginia School of Medicine (Charlottesville, USA).

### Socioeconomic status survey

We adapted demographic and SES questions from the most recent DHS questionnaires [13]. Improved water and sanitation were based on World Health Organization definitions [14]. Site investigators reviewed questionnaires and identified items that were problematic in their sites. Each site approved a final list of questions and response categories and the associated data collection procedures. Final demographic questions focused on age and education of the head of household and child’s mother, as well as mother’s fertility history. The SES section assessed household assets, housing materials, water source and sanitation facilities, and ownership of land or livestock. The survey also included a question on monthly household income in local currency. The questionnaire was developed in English and translated into local languages as appropriate and back-translated for quality assurance.

### Anthropometry

Field workers measured the selected child aged 24 to 60 months for height and weight in each participating household. Trained field staff used a locally produced platform with sliding headboard to measure standing height to the nearest 0.1 cm. They used digital scales to measure weight to the nearest 100 grams. We used the 2006 World Health Organization Multi-Country Growth Reference Study (WHO MGRS) to calculate height-for-age Z-scores (HAZ). Based on these standards, we defined stunting as a HAZ less than two standard deviations below the global median [15].

### Biostatistical methods

Our statistical analyses comprised two phases. First, we identified the best approach to selecting and weight household assets as a proxy for wealth. Second, we combined our wealth measure with other relevant variables to form a more complete measure of household SES. In both phases we assessed the associations between SES/wealth measures and child HAZ for two reasons: 1) we were interested in directly comparing the predictive power of wealth/SES measures, and 2) assessing associations between a construct of interest and other constructs that are believed to be related theoretically or empirically is one way of assessing construct validity [16]. We chose HAZ rather than weight-for-height because the former is a better measure of chronic deprivation, while the latter commonly indicates a composite of acute and chronic deprivation [3]. In both phases of analyses we were guided by a desire to identify the simplest valid measure of wealth or SES in terms of variables and computation required.

We compared four approaches to selecting and weighting indicators to measure household wealth: maternal education, PCA, MPI, and a novel variable selection method based on the use of conditional random forests [17]. We used maternal education as a baseline to assess the added value of assets beyond this commonly used proxy for household wealth [18,19]. Maternal education was constructed as a simple continuous measure of years of education completed by the child’s mother at the time of the survey. To construct the PCA-based SES index, we first selected a subset of dichotomous indicators, including assets, housing materials, and facilities, using Cronbach’s coefficient alpha. PCA was then conducted on the tetrachoric correlation matrix of selected indicators Additional file 1: Table S1, and we used the first principal component as the SES score for each household [20]. The MPI index, adapted from the UNDP approach based on available data, included the following indicators: maternal education (years of schooling); health (any child has died); and standard of living (electricity, water, sanitation, flooring, cooking fuel, and ownership of more than one of seven assets). Although the UNDP includes child nutritional status, we excluded this variable because it was our outcome of interest. We weighted these three areas equally to create a household wealth score [12]. Random forests (RF) are an expansion on classification trees using bootstrapping methods to generate multiple trees [17]. The RF approach to measuring wealth used the same initial indicators as the PCA method to ensure comparability of results Additional file 1: Table S1, i.e., so that differences in predictive power could be attributed to the method rather than the selection of assets. We used unsupervised learning with random forests to calculate conditional variable importance using the cforest package in R, which produces a variable importance rank in terms of their predictive value of a specified outcome (i.e., HAZ). Ownership of a subset of indicators was summed to create household wealth scores.

We then compared the three approaches (PCI, MPI, and RF) with maternal education to measure household wealth and the strength association with HAZ. The following criteria were used to compare the three wealth measurement approaches vs. maternal education: 1) leave-one-out cross-validation; 2) coefficient of determination (R^2^) values based on linear regression models with each wealth measure as the predictor and indicator variables for each site; and 3) scaled effect sizes from the same regression models. Leave-one-out cross-validation uses all observations except one to identify important variables for classification, while the remaining observation is used as the test set to measure the predictive error. This process is repeated using each observation as the test set to calculate the mean squared error (MSE) [17]. The approach with the smallest MSE predicts HAZ the most accurately. We also calculated 10-fold cross validation (results not shown), which produced similar findings to leave-one-out cross-validation. The coefficient of determination R^2^ represents the proportion of variability explained by a statistical model. The approach with the largest coefficient of determination captures the most variability in HAZ [21]. The effect size represents the estimated change in HAZ for each one-unit change in household wealth. Since the scales of each approach vary, we compared the effect of a 25% increase in each measure of household wealth.

We then examined associations between each wealth measurement approach and monthly household income. We converted household income to USD using January 1, 2010 exchange rates. Given the expected association between household wealth and income, these analyses provided evidence of the construct validity of each approach to measuring household wealth [22]. Based on the cumulative evidence from these analyses, we selected one approach to measuring household wealth.

The second phase of our analyses sought to incorporate several aspects of SES: access to improved **W**ater and sanitation, the selected approach to measuring household wealth (**A**ssets), **M**aternal education, and **I**ncome (i.e. the WAMI index). We included improved water and sanitation in response to guidance that SES measures should be based on hypothesized causal pathways in a study [23]. We then examined the predictive power of this composite measure of household SES relative to HAZ using the criteria described above. We used R 2.10.1 (http://www.r-project.org) and STATA 12.1 (STATA Corp., College Station, USA) for statistical analysis.

## Results

### Study sample characteristics

**Figure 1:**
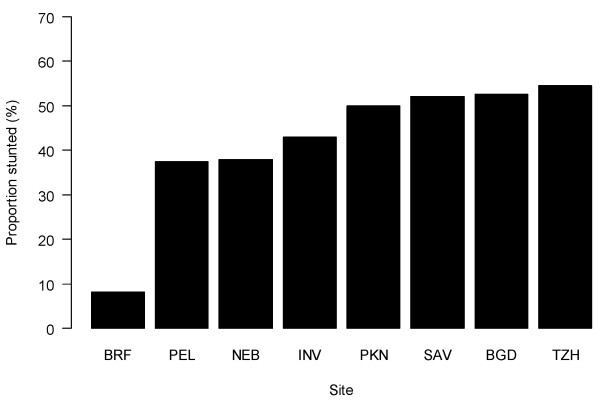
**Proportion of stunted children (height-for-age < -2 Z-scores) aged 24 to 60 months by study site.** Study sites are: Dhaka, Bangladesh (BGD); Fortaleza, Brazil (BRF); Vellore, India (INV); Bhaktapur, Nepal (NEB); Naushahro Feroze, Pakistan (PKN); Loreto, Peru (PEL); Venda, South Africa (SAV); and Haydom, Tanzania (TZH).

We surveyed a total of 800 households across all sites. One child had missing anthropometry and 10 were excluded for extreme HAZ values, resulting in a final sample size of 789 households (99% of original sample). All remaining observations had complete data on the variables used for these analyses. Mean age of sampled children was 41 months (SD = 10.4); 52% of children were male, ranging from 59% in Tanzania to 44% in Pakistan. Overall, 42% of children were stunted, ranging from 8% to 55% by site (Figure 1). Differences across sites were evidenced by variations in maternal education (from 3.3 years in Pakistan to 10.1 years in South Africa) and proportion with a bank account (from 2% in Tanzania to 76% in South Africa) (see Table 2). Nearly all households, with the exception of those in Tanzania, reported improved water and sanitation. Bivariate associations between stunting and either demographic or wealth indicators (e.g., age, water source, sanitation facility, maternal education, separate kitchen, and people per room) demonstrated the expected associations (Table 3).

**Table 2:** Selected socioeconomic characteristics of households overall and by country (n = 789)

		***Overall***	***Bangladesh***	***Brazil***	***India***	***Nepal***	***Pakistan***	***Peru***	***South Africa***	***Tanzania***
	***Sample size***	**789**	**99**	**98**	**100**	**100**	**98**	**99**	**96**	**99**
*Wealth indicators*	Separate room for a kitchen	50%	10%	87%	23%	73%	27%	85%	74%	21%
	Household bank account	31%	23%	21%	10%	62%	39%	15%	76%	2%
	Mattress	58%	66%	98%	1%	99%	13%	82%	66%	39%
	Refrigerator	31%	12%	88%	3%	24%	27%	21%	78%	0%
	TV	63%	55%	97%	69%	90%	61%	68%	68%	0%
	People per room (mean)	1.7	3.7	1.3	3.9	2.5	5.5	1.6	1.2	1.7
	Table	57%	29%	86%	21%	65%	50%	100%	74%	33%
	Chair or bench	61%	37%	94%	59%	68%	21%	95%	95%	16%
*Education*	Mean maternal education (years)	6.4	3.7	7.8	6.7	6.6	3.3	7.8	10.1	5.3
*Hygiene*	Improved water source	86%	100%	100%	100%	98%	100%	98%	65%	28%
	Improved sanitation facility	72%	100%	100%	37%	100%	74%	84%	84%	1%

Wealth indicators are listed in terms of variable importance (highest to lowest) in predicting height-for-age Z-score, based on the random forests approach.

**Table 3:** Relationship between selected indicators and child stunting

	**N**	**% stunted**	**p-value**
Sex			
Male	407	42.1	0.95
Female	382	41.9	
Age			
24-35 months	284	41.2	0.01
36-47 months	243	49.0	
48-60 months	262	36.3	
Water source			
Not improved	109	58.7	<0.001
Improved	680	39.3	
Sanitation facility			
Not improved	218	49.5	<0.01
Improved	571	39.1	
Maternal education			
None	135	57.0	<0.001
1-5 years	174	43.1	
> 5 years	480	37.3	
Separate kitchen			
No	396	51.0	<0.001
Yes	393	32.8	
People per room			
< 2	433	35.3	<0.001
≥ 2	356	50.0	

### Household wealth measurement

Drawing on the Cronbach’s alpha results showing internal consistency and reliability, we selected 16 assets to use in the PCA and RF analyses (final alpha = 0.86). We eliminated variables with low variation between households (defined as fewer than 10% of households in one category), and variables, the inclusion of which led to a significant drop in internal consistency reliability. The final 16 assets included were: iron, mattress, chair, sofa, cupboard, table, radio, computer, TV, sewing machine, mobile phone, fridge, bank account, separate kitchen, electricity, and people per room. Based on an approach similar to the scree plot used in PCA, where the magnitude of the change between each value is used to select a cutoff point, we ordered the 16 variables by their importance and selected the top eight for the RF measure. We compared the three wealth measurement approaches to mother’s education in terms of predictive value (MSE), explained variability (R^2^), and effect size (Table 4). The RF and PCA approaches performed better in terms of predictive value, explained variability, and effect size, and all of the wealth measurement approaches performed better than maternal education.

**Table 4:** Results comparing three wealth measurement approaches and mother’s education in terms of value in predicting child HAZ (n = 789)

	**Wealth method 1: maternal education**	**Wealth method 2: principal components analysis**	**Wealth method 3: multidimensional poverty Index**	**Wealth method 4: asset selection by random forests**	**Full SES Measure: Water and sanitation, Assets, Maternal education, Income (WAMI) index**
Mean squared error (MSE) from leave-one-out cross validation	1.39	1.37	1.39	1.37	1.37
Adjusted R^2^	18.67%	19.98%	18.89%	19.86%	20.19%
Effect size (95% CI) Change in HAZ associated with a 25% increase in wealth score	0.123 (0.029-0.217) *p = 0.01*	0.290 (0.164-0.416) *p < 0.001*	0.149 (0.061-0.237) *p = 0.002*	0.220 (0.121-0.319) *p < 0.001*	0.384 (0.222-0.546) *p < 0.001*
Number of variables	1	16 variables summarized into 1	14 variables summarized into 1	Selected 8 out of 16 variables, and summarized these 8 variables into 1	Summarized 12 variables into 1

**Figure 2:**
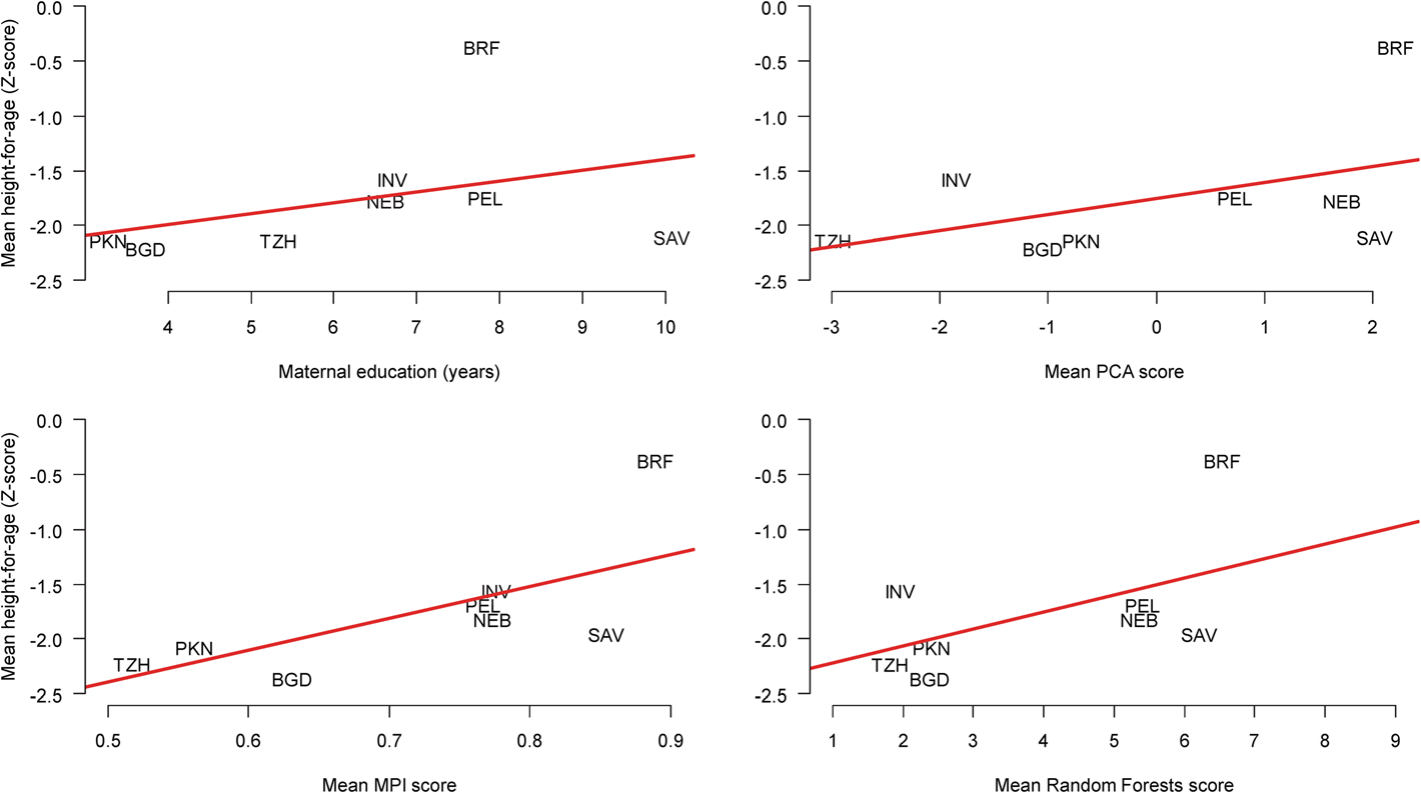
**Relationship between height-for-age of children aged 24 to 60 months and four different types of socioeconomic status indices.** Each data point represents the average score for either height-for-age or each socioeconomic status index at each study site. The red line indicates a linear regression fit of the relationship between height-for-age and socioeconomic status indices using site as the unit of analysis. Study sites are: Dhaka, Bangladesh (BGD); Fortaleza, Brazil (BRF); Vellore, India (INV); Bhaktapur, Nepal (NEB); Naushahro Feroze, Pakistan (PKN); Loreto, Peru (PEL); Venda, South Africa (SAV); and Haydom, Tanzania (TZH).

All tested wealth measures were significantly associated with HAZ, with slightly stronger associations for the PCA and RF measures. The ranking of mean wealth score by site followed a similar pattern across measures: a low group including the Tanzania, Pakistan, and Bangladesh sites; a middle group including the India, Peru, and Nepal sites; and a higher group including the South Africa and Brazil sites. Mean HAZ in Brazil was higher than would be predicted by the regression line, while the opposite was true for South Africa (Figure 2).

### Income and wealth

**Figure 3:**
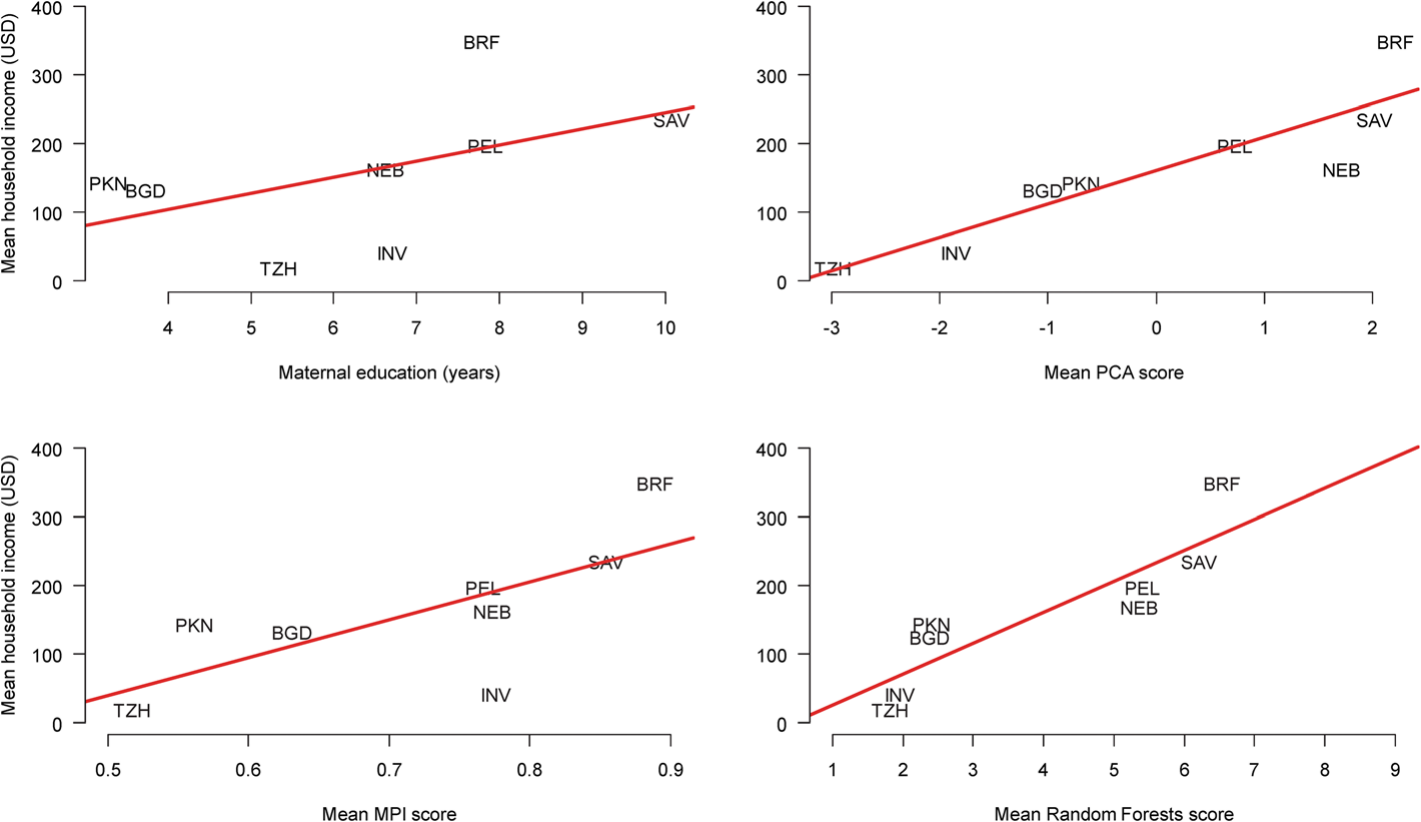
**Relationship between household income and four different types of socioeconomic status indices.** Each data point represents the average household income or average score for each socioeconomic status index at each study site. The red line indicates a linear regression fit of the relationship between household income and socioeconomic status indices using site as the unit of analysis. Study sites are: Dhaka, Bangladesh (BGD); Fortaleza, Brazil (BRF); Vellore, India (INV); Bhaktapur, Nepal (NEB); Naushahro Feroze, Pakistan (PKN); Loreto, Peru (PEL); Venda, South Africa (SAV); and Haydom, Tanzania (TZH).

Each wealth measure was also significantly associated with monthly household income (Figure 3). These associations were strongest for the PCA and RF approaches to measuring wealth. The ranking of sites by mean monthly household income followed a similar pattern to wealth overall, with some notable departures. For example, the Pakistan site ranked lowest in terms of mean number of years of mother’s education, but ranked fifth out of eight sites in terms of monthly income. When comparing wealth measured by PCA with household income, however, the rankings were nearly identical. When wealth was measured using the RF method, the South Africa and Nepal sites had nearly the same mean wealth score, but the South African site ranked higher in terms of mean monthly income. The associations between wealth measure and monthly income in each site provide evidence of the construct validity of each measure of wealth, while the changes in site rankings demonstrate the importance of including both wealth and income in a complete measure of household SES.

### Choice of wealth measure

We chose the RF approach to measuring wealth. We ruled out the approach of using maternal education alone because it performed poorly relative to the other measures, it was inconsistent with our theoretical understanding of wealth, and availability of education to women is dependent on societal values and investments, which is highly heterogeneous across low- and middle-income countries such as those in these studies. PCA and RF performed better than MPI in terms of predictive validity and variation explained in HAZ (Table 4). Both the PCA and RF approaches require statistical calculations. PCA requires use of different weights for each variable that have no inherent meaning, whereas RF can be used initially to identify important variables, which can then be combined using a simple approach. While the PCA approach retains all variables, potentially resulting in the inclusion of variables that are irrelevant in some study sites, the RF approach keeps only the variables that are most closely related to the outcome of interest.

### Development of an integrated socioeconomic status index

**Figure 4:**
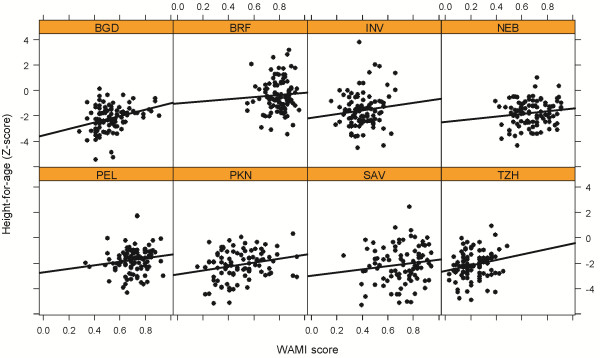
**Scatterplot of height-for-age and WAMI index score stratified by study site.** The black line represents a linear regression fit of the relationship between height-for-age and WAMI index score at each site. Study sites are: Dhaka, Bangladesh (BGD); Fortaleza, Brazil (BRF); Vellore, India (INV); Bhaktapur, Nepal (NEB); Naushahro Feroze, Pakistan (PKN); Loreto, Peru (PEL); Venda, South Africa (SAV); and Haydom, Tanzania (TZH).

We formulated a complete index of household SES, including the following components: access to improved **W**ater and sanitation, wealth measured by a set of eight **A**ssets, **M**aternal education, and monthly household **I**ncome (i.e. WAMI index). In Table 5, we show our approach to combining these four components into a complete measure, and in Figure 4 we show associations between the WAMI index and HAZ. We compare the WAMI index with the wealth-only measures in Table 4. While the WAMI index is comparable to wealth alone in terms of predictive value and variation explained, there is a notable difference in the effect size. A 25% difference in the WAMI score is positively associated with a difference of 0.38 SD in HAZ (95% CI 0.22 to 0.55). In contrast, a 25% difference in wealth score alone (RF approach) is only associated with a 0.22 SD difference in HAZ (95% CI 0.12 to 0.32).

**Table 5:** Calculation of the Water/sanitation, Assets, Maternal education, and Income (WAMI) index

	**Description**	**Range**
***Water/sanitation***	Using World Health Organization definitions of access to improved water and improved sanitation, households with access to improved water or improved sanitation are assigned a score of 4 for each. Households without access to improved water or improved sanitation are assigned a score of 0 for each. These scores were summed.	0-8
***Assets***	Eight priority assets were selected using random forests with HAZ as the outcome. For each asset, households were assigned a 1 if they have the asset and 0 if they do not have the asset. These scores were summed.	0-8
***Maternal education***	Each child’s mother provided the number of years of schooling she had completed, ranging from 0 to 16 years. This number was divided by 2.	0-8
***Income***	Monthly household income was converted to US dollars using the exchange rate from January 1, 2010. Income was divided into octiles using the following scores and cutoffs: 1 (0–26), 2 (26.01-47), 3 (47.01-72), 4 (72.01-106), 5 (106.01-135), 6 (135.01-200), 7 (200.01-293), 8 (293+).	0-8
***TOTAL***	Scores in water and sanitation, assets, mother’s education, and income were summed then divided by 32.	0-1

## Discussion

We compared three approaches to measuring household wealth with maternal education and selected the random forests approach due to its consistent association with HAZ and simplicity of use and interpretation of selected assets relative to PCA. Although maternal education was the simplest approach as it only required one variable, it did not reflect theoretical models of household SES, an important consideration in choice of variables [24]. In addition, maternal education is strongly influenced by culture. Our data support previous evidence that, while wealth and education are important components of SES, in many settings they do not measure the same exposures [25].

We then combined our selected wealth measure with access to improved water and sanitation, maternal education, and household income to form a complete SES measure. Krieger and colleagues [26] argue that the term SES “blurs distinctions between two different aspects of socioeconomic position: (a) actual resources, and (b) status, meaning prestige- or rank-related characteristics.” We use the term SES rather than socioeconomic position because the latter is intended to explicitly include prestige-based measures, as linked to social class. Our measure focuses only on the actual resources in a household because we did not collect prestige-based indicators in our study. Hackman and Farah [25] emphasize the importance of a clear conceptual framework driving the measurement of socioeconomic status. In the MAL-ED study, which focuses on the relationships between enteric infections in infancy and subsequent growth and cognitive development, access to improved water and sanitation sources are important risk factors that are closely related to SES. Our final measure requires 12 variables, most of which are easily collected across diverse settings, which is a requirement in order for an SES measure to be applicable in a multicountry study.

Compared to maternal education alone, as well as more complete measures of wealth, the WAMI index demonstrated a significantly stronger association with HAZ across our eight study sites. These results provide evidence of the importance of using a robust measure of household SES, even as an adjustment factor, rather than a measure of wealth or education alone. Based on analyses of a nationally representative sample in the United States, Braveman and colleagues [25] found that, depending on the choice of income, education, or both as a control for SES, the ranking of racial groups by odds of poor health changed dramatically. Many studies also seek to simplify measurement of SES by identifying the variables that are most strongly associated with their outcomes. Daly and colleagues [27] found that wealth and income indicators were more strongly associated with mortality than education and occupation indicators in a United States cohort. In settings where collection of income data is not feasible, an expanded measure of SES including wealth, education, and water and sanitation is still a significant improvement over wealth or maternal education alone (results not shown).

Our study has a number of important strengths. It includes data from households from eight country sites located in South Asia, sub-Saharan Africa, and Latin America to derive a multicountry index. The experienced staff in these eight sites used a standardized protocol with identical questionnaires to collect demographic and SES information. The survey included extensive questions related to SES, due to an a priori interest in identifying an appropriate approach to measuring this construct. Our analyses systematically compared both commonly used and new approaches to measuring wealth and SES. Our final selection of a measure of socioeconomic status balances statistical and theoretical strength with feasibility in a field research setting.

The results of this study should be considered in light of some limitations. Although the MAL-ED study sites are located in eight diverse country settings, they are not nationally representative samples. Therefore, the results cannot be generalized to national comparisons or compared to country-level indices such as the DHS. More broadly, the resulting components of an ideal measure of SES are likely to vary across settings and study objectives. Consistencies across our study sites indicate that it is possible to identify measures of SES that are relevant in diverse settings. However, the actual variables that form an ideal measure will be informed by the study populations, settings, and research questions. Rather than selecting indicators to be used by all studies, our findings are intended to demonstrate the importance of developing a measure of socioeconomic status that is theoretically sound and contextually relevant, especially in multisite or multicountry studies. There is no gold standard measure of socioeconomic status against which to compare our proposed measure, particularly in a multicountry study setting. However, associations between the WAMI index and HAZ demonstrate construct validity of the measure, since we expect these constructs to be associated theoretically. Another limitation of our study is that we do not have a measure of occupation. Much of the work on socioeconomic status that includes occupation as a key component is based on research in high-income settings. In many low-income settings, this concept does not distinguish between households as readily, either due to homogeneity or instability of income sources. Alternatives include caste or religious group. While we collected data on these groupings, we did not feel that they were sufficiently comparable across the eight study sites. Similarly, indicators of prestige or rank-related characteristics have been included in measures of socioeconomic position [26], but these are unavailable in our dataset. With the exception of data on access to electricity, our available SES indicators were measured at the household level, rather than the community level. Previous research has shown that the addition of community-level variables to SES measures can be helpful in exploring trends and inequalities in health outcomes [28].

In summary, novel classification approaches such as random forests provide an alternative to the more widely used PCA for the measurement of household wealth. However, assets alone are not sufficient to capture the full domain of socioeconomic status. We developed a simplified socioeconomic index that combines measures of improved water and sanitation, assets, maternal education, and household income that may be applicable to a multicountry setting. We believe that this measure is an improvement over the commonly used PCA-based wealth measurement approach for several reasons: 1) It is a robust measure that more fully reflects a theoretical understanding of SES; 2) it reduces the data collection burden by highlighting a priority set of indicators for measurement, in contrast to commonly used PCA approaches, which require collecting data on a full set of indicators, even if some are irrelevant; and 3) it is computationally simple to apply, once the priority assets have been selected using the random forests technique. With further validation, this simplified WAMI index may provide a standardized approach for adjustment across diverse study populations.

## Competing interests

The authors have no competing interests to declare.

## Authors’ contribution

SP and WC contributed equally to the conception, design and analysis of data, and interpretation of findings. SP led the writing of the manuscript. SP, JS, MM, MG, ZB, TA, AS, PB, SJ, GK, MK, AL, PS, ES, and WC contributed equally to study design and data acquisition. All authors read and approved the final manuscript. WC had ultimate oversight over the study design, data analysis, and writing of this manuscript.

## Supplementary Material

Additional file 1: Table S1 Variables included in initial PCA and random forests analyses. Sites are listed from left to right, starting with the highest mean WAMI score (Brazil) and ending with the lowest mean WAMI score (Tanzania). Click here for file
