# Supplementary material for: Measuring socioeconomic status in multicountry studies: results from the eight-country MAL-ED study
Source: Popul Health Metr. 2014 Mar 21;12:8. doi: 10.1186/1478-7954-12-8 (PMC4234146; doi:10.1186/1478-7954-12-8)
Supplement: Additional file 1: Table S1 — Variables included in initial PCA and random forests analyses. Sites are listed from left to right, starting with the highest mean WAMI score (Brazil) and ending with the lowest mean WAMI score (Tanzania). [file 1478-7954-12-8-S1.doc]

**Additional file 1: Table S1.** Mean (SD) values for pool of indicators included in initial PCA and random forests analyses. Sites are listed from left to right, starting with the highest mean WAMI score (Brazil) and ending with the lowest mean WAMI score (Tanzania).

| ***% households who own each asset*** | **Brazil**  **(n=98)** | **Peru**  **(n=99)** | **S. Africa**  **(n=96)** | **Nepal**  **(n=100)** | **Bangladesh**  **(n=99)** | **Pakistan**  **(n=98)** | **India**  **(n=100)** | **Tanzania**  **(n=99)** | **Total**  **(n=789)** |
| --- | --- | --- | --- | --- | --- | --- | --- | --- | --- |
| Bank account | 21 | 15 | 76 | 62 | 23 | 39 | 10 | 2 | 31 |
| Chair or bench | 94 | 95 | 95 | 68 | 38 | 21 | 59 | 16 | 61 |
| Computer | 11 | 3 | 12 | 20 | 0 | 4 | 5 | 0 | 7 |
| Crowding (>2 people/room) | 16 | 25 | 10 | 46 | 95 | 98 | 91 | 31 | 52 |
| Cupboard | 76 | 75 | 76 | 92 | 63 | 45 | 31 | 4 | 58 |
| Electricity | 99 | 85 | 94 | 99 | 100 | 98 | 97 | 0 | 84 |
| Iron | 56 | 50 | 83 | 63 | 11 | 73 | 8 | 30 | 47 |
| Kitchen | 87 | 85 | 74 | 73 | 10 | 27 | 23 | 21 | 50 |
| Refrigerator | 88 | 21 | 78 | 24 | 12 | 27 | 3 | 0 | 31 |
| Mattress | 98 | 82 | 66 | 99 | 66 | 13 | 1 | 39 | 58 |
| Mobile phone | 81 | 31 | 96 | 96 | 63 | 68 | 53 | 54 | 68 |
| Radio | 74 | 55 | 82 | 48 | 11 | 12 | 2 | 46 | 41 |
| Sewing machine | 9 | 12 | 13 | 43 | 5 | 38 | 3 | 1 | 16 |
| Sofa | 47 | 4 | 51 | 42 | 3 | 14 | 5 | 2 | 21 |
| Table | 86 | 100 | 74 | 65 | 29 | 50 | 21 | 33 | 57 |
| TV | 97 | 68 | 68 | 90 | 55 | 61 | 69 | 0 | 63 |
